# Supplementary material for: Dynamics of embryonic stem cell differentiation inferred from single-cell transcriptomics show a series of transitions through discrete cell states
Source: eLife. 2017 Mar 15;6:e20487. doi: 10.7554/eLife.20487 (PMC5352225; doi:10.7554/eLife.20487)
Supplement: Figure 1—source data 1. — DOI: http://dx.doi.org/10.7554/eLife.20487.003 [file elife-20487-fig1-data1.docx]

**Figure 1 – Source Data 1: Differentiation conditions and duration of single cells sorted into seven 96-well plates**

|  | plate M1 | plate M2 | plate M3 | plate M5 | plate M6 | plate M7 | plate M8 |
| --- | --- | --- | --- | --- | --- | --- | --- |
| row A | Lif2i | Day 1 ChAct | Day 2+2 PD0+LDN | Day 1 ChAct | Day 3+1 ChA+LDN | Day 3+2 ChA+LDN | Day 3+2 ChA+LDN |
| row B | Lif2i | Day 1 ChAct | Day 2+2 PD0+LDN | Day 1 ChAct | Day 3+1 ChA+LDN | Day 3+2 ChA+LDN | Day 3+2 ChA+LDN |
| row C | Day 1 PD0 | Day 3 ChAct | Day 2+2 PD0+LDN | Das 3 ChAct | Day 3+1 ChA+LDN | Day 3+2 ChA+LDN | Day 3+2 ChA+LDN |
| row D | Day 1 PD0 | Day 3 ChAct | Day 2+2 PD0+LDN | Day 3 ChAct | Day 3+1 ChA+LDN | Day 3+2 ChA+LDN | Day 3+2 ChA+LDN |
| row E | Day 2 PD0 | Day 2+1 PD+LDN | Day 2+2 PD0+Bmp | Day 2+1 PD+LDN | Day 3+1 ChA+Bmp | Day 3+2 ChA+Bmp | Day 3+2 ChA+Bmp |
| row F | Day 2 PD0 | Day 2+1 PD+LDN | Day 2+2 PD0+Bmp | Day 2+1 PD+LDN | Day 3+1 ChA+Bmp | Day 3+2 ChA+Bmp | Day 3+2 ChA+Bmp |
| row G | Day 2 ChAct | Day 2+1 PD+Bmp | Day 2+2 PD0+Bmp | Day 2+1 PD+Bmp | Day 3+1 ChA+Bmp | Day 3+2 ChA+Bmp | Day 3+2 ChA+Bmp |
| row H | Day 2 ChAct | Day 2+1 PD+Bmp | Day 2+2 PD0+Bmp | Day 2+1 PD+Bmp | Day 3+1 ChA+Bmp | Day 3+2 ChA+Bmp | Day 3+2 ChA+Bmp |
