# Supplementary material for: Dynamics of embryonic stem cell differentiation inferred from single-cell transcriptomics show a series of transitions through discrete cell states
Source: eLife. 2017 Mar 15;6:e20487. doi: 10.7554/eLife.20487 (PMC5352225; doi:10.7554/eLife.20487)
Supplement: Figure 2—source data 1. — DOI: http://dx.doi.org/10.7554/eLife.20487.006 [file elife-20487-fig2-data1.docx]

**Figure 2 – Source Data 1: Plate and well id’s of cells belonging to each cluster**

| C_0_ | C_1_ | C_2_ | C_3_ | C_4_ | C_5_ | C_6_ | C_7_ | C_8_ |
| --- | --- | --- | --- | --- | --- | --- | --- | --- |
| M1_A01 | M1_F01 | M2_C01 | M2_F01 | M8_B01 | M3_C01 | M3_H01 | M5_G04 | M8_F01 |
| M1_D01 | M1_E01 | M2_D01 | M2_H01 | M8_B02 | M3_D01 | M3_H02 | M6_B01 | M8_E01 |
| M1_A02 | M1_E02 | M2_D02 | M2_E03 | M8_A02 | M3_D02 | M3_G01 | M6_A01 | M8_E02 |
| M1_B03 | M1_E03 | M2_C02 | M2_E04 | M8_B03 | M3_C02 | M3_G02 | M6_B02 | M8_E03 |
| M1_A07 | M1_E04 | M2_D03 | M2_H03 | M8_A07 | M3_C03 | M3_H04 | M6_F01 | M8_F02 |
| M1_C02 | M1_H03 | M2_D04 | M2_G02 | M8_A03 | M3_C04 | M3_H05 | M6_H01 | M8_F03 |
| M1_A03 | M1_G01 | M2_D07 | M2_F03 | M8_A04 | M3_D07 | M3_G03 | M6_H02 | M8_E05 |
| M1_A04 | M1_G02 | M2_C06 | M2_H05 | M8_B04 | M3_C05 | M3_H06 | M6_C01 | M8_E06 |
| M1_B04 | M1_F02 | M2_C08 | M2_G03 | M8_A05 | M3_C06 | M3_G04 | M6_E03 | M8_F07 |
| M1_D05 | M1_F03 | M2_C09 | M2_G04 | M8_B05 | M3_C07 | M3_G05 | M6_D02 | M8_F08 |
| M1_A05 | M1_G03 | M2_D08 | M2_F04 | M8_B06 | M3_C08 | M3_G06 | M6_B03 | M8_E07 |
| M1_D06 | M1_G04 | M2_D09 | M2_E05 | M8_A06 | M3_D08 | M3_H07 | M6_A07 | M8_E11 |
| M1_D07 | M1_F04 | M2_D10 | M2_G05 | M8_B07 | M3_D09 | M3_H08 | M6_C02 | M8_F10 |
| M1_B05 | M1_E05 | M2_C10 | M2_E06 | M8_B08 | M3_D10 | M3_G07 | M6_G01 | M8_F11 |
| M1_B06 | M1_G06 | M2_C11 | M2_H08 | M8_B09 | M3_C11 | M3_H09 | M6_G02 |  |
| M1_A06 | M1_F05 | M2_D11 | M2_G07 | M8_A08 |  | M3_H10 | M6_F02 |  |
| M1_C05 | M1_F06 | M5_D01 | M2_H09 | M8_A10 |  | M3_G09 | M6_F03 |  |
| M1_B07 | M1_H09 | M5_D02 | M2_F07 | M8_A11 |  | M3_H11 | M6_D04 |  |
| M1_C07 | M1_G08 | M5_C02 | M2_E07 | M8_B10 |  | M3_G10 | M6_H05 |  |
| M1_C08 | M1_F07 | M5_C03 | M2_E08 | M8_B11 |  | M3_G11 | M6_A04 |  |
| M1_C09 | M1_H10 | M5_D04 | M2_E09 |  |  |  | M6_D05 |  |
| M1_B08 | M1_E07 | M5_D05 | M2_H11 |  |  |  | M6_F04 |  |
| M1_B09 | M1_E09 | M5_D06 | M2_E11 |  |  |  | M6_A05 |  |
| M1_A10 | M1_H11 | M5_D07 | M2_F10 |  |  |  | M6_D06 |  |
| M1_D08 | M1_E10 | M5_C05 | M2_G10 |  |  |  | M6_E05 |  |
| M1_D10 | M1_E11 | M5_C06 | M2_G11 |  |  |  | M6_G05 |  |
| M1_C10 | M1_F10 | M5_C07 | M5_F01 |  |  |  | M6_F05 |  |
| M1_C11 | M1_G10 | M5_C09 | M5_E01 |  |  |  | M6_F06 |  |
| M1_D11 | M1_G11 | M5_D08 | M5_H02 |  |  |  | M6_B05 |  |
| M2_A01 |  | M5_D09 | M5_E04 |  |  |  | M6_B06 |  |
| M2_B03 |  | M5_D10 | M5_F02 |  |  |  | M6_A06 |  |
| M2_A07 |  | M5_C10 | M5_G03 |  |  |  | M6_C05 |  |
| M2_A03 |  | M5_C11 | M5_H06 |  |  |  | M6_C06 |  |
| M2_A04 |  |  | M5_F04 |  |  |  | M6_C07 |  |
| M2_A05 |  |  | M5_E05 |  |  |  | M6_C08 |  |
| M2_B05 |  |  | M5_E06 |  |  |  | M6_H09 |  |
| M2_B06 |  |  | M5_G06 |  |  |  | M6_H10 |  |
| M2_A06 |  |  | M5_F06 |  |  |  | M6_C09 |  |
| M2_B07 |  |  | M5_H08 |  |  |  | M6_B09 |  |
| M2_B09 |  |  | M5_F07 |  |  |  | M6_A08 |  |
| M2_A08 |  |  | M5_F08 |  |  |  | M6_A09 |  |
| M2_A10 |  |  | M5_H10 |  |  |  | M6_A10 |  |
| M2_A11 |  |  | M5_F09 |  |  |  | M6_E09 |  |
| M2_B10 |  |  | M5_E08 |  |  |  | M6_D08 |  |
| M2_B11 |  |  | M5_E10 |  |  |  | M6_E10 |  |
| M5_B01 |  |  | M5_E11 |  |  |  | M6_A11 |  |
| M5_A01 |  |  |  |  |  |  | M6_D09 |  |
| M5_A04 |  |  |  |  |  |  | M6_D10 |  |
| M5_B04 |  |  |  |  |  |  | M6_C10 |  |
| M5_A05 |  |  |  |  |  |  | M6_B10 |  |
| M5_B05 |  |  |  |  |  |  | M6_G10 |  |
| M5_B06 |  |  |  |  |  |  | M6_C11 |  |
| M5_A06 |  |  |  |  |  |  | M6_B11 |  |
| M5_B07 |  |  |  |  |  |  | M8_G01 |  |
| M5_A08 |  |  |  |  |  |  |  |  |
| M5_A10 |  |  |  |  |  |  |  |  |
| M5_A11 |  |  |  |  |  |  |  |  |
