# Supplementary material for: Dynamics of embryonic stem cell differentiation inferred from single-cell transcriptomics show a series of transitions through discrete cell states
Source: eLife. 2017 Mar 15;6:e20487. doi: 10.7554/eLife.20487 (PMC5352225; doi:10.7554/eLife.20487)
Supplement: Figure 2—source data 2. — DOI: http://dx.doi.org/10.7554/eLife.20487.007 [file elife-20487-fig2-data2.docx]

**Figure 2 – Source Data 2: Triplet probabilities of final tree.**

| triplet | | | probabilities for prior odds p(β_i=1)/p(β_i=0) = 1E-5 | | | | number of non-null topologies with prob > 0.6 | most likely topology | probability at max |
| --- | --- | --- | --- | --- | --- | --- | --- | --- | --- |
| A | B | C | p(**A**\|{g},{C}) | p(**B**\|{g},{C}) | p(**C**\|{g},{C}) | p(**0**\|{g},{C}) |  |  |  |
| C0 | C1 | C3 | 3.05E-34 | 1.00E+00 | 1.01E-89 | 2.99E-04 | 1 | C1 | 1.000 |
| C0 | C1 | C8 | 1.44E-15 | 1.00E+00 | 6.00E-218 | 6.32E-07 | 1 | C1 | 1.000 |
| C0 | C1 | C5 | 5.33E-31 | 1.00E+00 | 5.37E-101 | 4.14E-05 | 1 | C1 | 1.000 |
| C0 | C1 | C6 | 9.52E-27 | 1.00E+00 | 1.89E-103 | 4.48E-04 | 1 | C1 | 1.000 |
| C0 | C1 | C7 | 4.14E-05 | 9.99E-01 | 1.48E-181 | 1.27E-03 | 1 | C1 | 0.999 |
| C0 | C1 | C4 | 3.90E-24 | 1.00E+00 | 6.95E-131 | 1.54E-04 | 1 | C1 | 1.000 |
| C0 | C1 | C2 | 9.20E-08 | 9.36E-01 | 5.13E-13 | 6.44E-02 | 1 | C1 | 0.967 |
| C0 | C3 | C8 | 1.16E-179 | 5.78E-10 | 1.03E-26 | 1.00E+00 | 0 | null | 1.000 |
| C0 | C3 | C5 | 3.67E-253 | 5.41E-01 | 4.59E-01 | 6.99E-08 | 1 | C3 | 0.613 |
| C0 | C3 | C6 | 1.53E-181 | 8.72E-01 | 1.28E-01 | 2.99E-07 | 1 | C3 | 0.976 |
| C0 | C3 | C7 | 1.31E-83 | 1.40E-07 | 6.99E-47 | 1.00E+00 | 1 | C3 | 0.798 |
| C0 | C3 | C4 | 2.10E-120 | 1.97E-17 | 3.87E-47 | 1.00E+00 | 0 | null | 1.000 |
| C0 | C3 | C2 | 2.01E-39 | 4.21E-57 | 9.94E-01 | 5.76E-03 | 1 | C2 | 0.996 |
| C0 | C8 | C5 | 4.77E-141 | 5.74E-01 | 5.99E-29 | 4.26E-01 | 1 | C8 | 0.994 |
| C0 | C8 | C6 | 2.98E-122 | 8.46E-11 | 5.60E-01 | 4.40E-01 | 2 | C8 | 0.703 |
| C0 | C8 | C7 | 2.00E-171 | 4.47E-01 | 5.53E-01 | 9.15E-05 | 1 | C7 | 0.642 |
| C0 | C8 | C4 | 1.28E-255 | 5.46E-07 | 1.00E+00 | 1.46E-06 | 1 | C4 | 1.000 |
| C0 | C8 | C2 | 1.02E-133 | 1.47E-65 | 1.00E+00 | 5.12E-05 | 1 | C2 | 1.000 |
| C0 | C5 | C6 | 1.59E-170 | 6.60E-01 | 3.40E-01 | 1.21E-08 | 1 | C5 | 0.775 |
| C0 | C5 | C7 | 2.55E-66 | 5.38E-31 | 2.84E-58 | 1.00E+00 | 0 | null | 1.000 |
| C0 | C5 | C4 | 2.82E-93 | 3.36E-37 | 4.62E-39 | 1.00E+00 | 0 | null | 1.000 |
| C0 | C5 | C2 | 4.30E-21 | 3.94E-67 | 9.89E-01 | 1.09E-02 | 1 | C2 | 0.997 |
| C0 | C6 | C7 | 1.31E-48 | 1.12E-01 | 1.35E-24 | 8.88E-01 | 1 | C6 | 0.903 |
| C0 | C6 | C4 | 1.64E-82 | 1.21E-08 | 3.26E-38 | 1.00E+00 | 1 | C6 | 0.708 |
| C0 | C6 | C2 | 8.53E-13 | 1.57E-41 | 9.89E-01 | 1.14E-02 | 1 | C2 | 0.996 |
| C0 | C7 | C4 | 1.01E-165 | 5.13E-01 | 4.87E-01 | 3.53E-05 | 0 | C7 | 0.538 |
| C0 | C7 | C2 | 1.03E-93 | 1.79E-39 | 9.92E-01 | 7.56E-03 | 1 | C2 | 0.997 |
| C0 | C4 | C2 | 6.05E-126 | 5.07E-03 | 9.92E-01 | 2.77E-03 | 1 | C2 | 0.999 |
| C1 | C3 | C8 | 8.60E-27 | 8.49E-07 | 4.52E-41 | 1.00E+00 | 0 | null | 1.000 |
| C1 | C3 | C5 | 2.70E-52 | 5.52E-01 | 4.35E-01 | 1.38E-02 | 1 | C3 | 0.660 |
| C1 | C3 | C6 | 2.10E-14 | 9.28E-01 | 1.86E-02 | 5.29E-02 | 1 | C3 | 0.980 |
| C1 | C3 | C7 | 8.89E-04 | 1.94E-06 | 2.60E-45 | 9.99E-01 | 1 | C1 | 0.800 |
| C1 | C3 | C4 | 2.62E-09 | 4.21E-14 | 1.28E-48 | 1.00E+00 | 1 | C1 | 0.705 |
| C1 | C3 | C2 | 8.47E-01 | 4.01E-46 | 1.61E-04 | 1.53E-01 | 1 | C1 | 0.879 |
| C1 | C8 | C5 | 8.88E-29 | 1.54E-01 | 7.67E-32 | 8.46E-01 | 1 | C8 | 0.820 |
| C1 | C8 | C6 | 4.48E-26 | 1.51E-17 | 3.46E-03 | 9.97E-01 | 1 | C6 | 0.816 |
| C1 | C8 | C7 | 6.05E-177 | 6.89E-01 | 4.28E-16 | 3.11E-01 | 1 | C8 | 0.949 |
| C1 | C8 | C4 | 1.26E-215 | 1.74E-16 | 9.97E-01 | 3.17E-03 | 1 | C4 | 0.998 |
| C1 | C8 | C2 | 1.80E-173 | 8.61E-53 | 9.96E-01 | 3.84E-03 | 1 | C2 | 0.998 |
| C1 | C5 | C6 | 9.57E-24 | 7.14E-01 | 2.77E-01 | 8.76E-03 | 1 | C5 | 0.882 |
| C1 | C5 | C7 | 1.12E-05 | 7.42E-23 | 9.43E-43 | 1.00E+00 | 1 | C1 | 0.874 |
| C1 | C5 | C4 | 8.72E-23 | 2.47E-40 | 3.55E-18 | 1.00E+00 | 0 | null | 1.000 |
| C1 | C5 | C2 | 1.52E-07 | 5.30E-62 | 9.22E-01 | 7.84E-02 | 1 | C2 | 0.945 |
| C1 | C6 | C7 | 1.13E-04 | 9.71E-04 | 7.74E-22 | 9.99E-01 | 0 | C6 | 0.578 |
| C1 | C6 | C4 | 1.71E-03 | 1.46E-13 | 1.58E-19 | 9.98E-01 | 1 | C1 | 0.709 |
| C1 | C6 | C2 | 7.99E-01 | 1.25E-36 | 1.58E-01 | 4.23E-02 | 1 | C1 | 0.897 |
| C1 | C7 | C4 | 1.04E-191 | 2.38E-09 | 9.91E-01 | 9.47E-03 | 1 | C4 | 0.995 |
| C1 | C7 | C2 | 2.67E-146 | 3.28E-16 | 9.50E-01 | 5.03E-02 | 1 | C2 | 0.971 |
| C1 | C4 | C2 | 3.29E-170 | 1.19E-01 | 5.87E-01 | 2.95E-01 | 1 | C2 | 0.605 |
| C3 | C8 | C5 | 1.02E-03 | 5.17E-86 | 9.89E-01 | 9.99E-03 | 1 | C5 | 0.994 |
| C3 | C8 | C6 | 9.91E-01 | 2.00E-55 | 5.49E-06 | 9.00E-03 | 1 | C3 | 0.993 |
| C3 | C8 | C7 | 6.83E-108 | 3.16E-02 | 6.22E-01 | 3.46E-01 | 1 | C7 | 0.798 |
| C3 | C8 | C4 | 3.67E-167 | 2.63E-20 | 9.88E-01 | 1.22E-02 | 1 | C4 | 0.988 |
| C3 | C8 | C2 | 7.72E-147 | 1.40E-12 | 5.53E-08 | 1.00E+00 | 1 | C2 | 0.890 |
| C3 | C5 | C6 | 3.30E-01 | 4.90E-01 | 1.72E-19 | 1.80E-01 | 0 | C5 | 0.583 |
| C3 | C5 | C7 | 1.45E-02 | 9.63E-01 | 8.46E-56 | 2.29E-02 | 1 | C5 | 0.980 |
| C3 | C5 | C4 | 1.92E-04 | 9.51E-01 | 2.21E-85 | 4.90E-02 | 1 | C5 | 0.955 |
| C3 | C5 | C2 | 2.15E-03 | 9.90E-01 | 1.64E-64 | 7.90E-03 | 1 | C5 | 0.994 |
| C3 | C6 | C7 | 9.19E-01 | 2.66E-04 | 5.57E-32 | 8.05E-02 | 1 | C3 | 0.941 |
| C3 | C6 | C4 | 6.98E-01 | 1.57E-01 | 6.29E-40 | 1.45E-01 | 1 | C3 | 0.824 |
| C3 | C6 | C2 | 7.89E-01 | 1.33E-01 | 1.54E-14 | 7.85E-02 | 1 | C3 | 0.920 |
| C3 | C7 | C4 | 1.04E-132 | 9.84E-01 | 3.90E-09 | 1.62E-02 | 1 | C7 | 0.987 |
| C3 | C7 | C2 | 1.86E-112 | 2.51E-07 | 9.97E-07 | 1.00E+00 | 1 | C2 | 0.792 |
| C3 | C4 | C2 | 3.36E-147 | 9.38E-01 | 3.31E-05 | 6.16E-02 | 1 | C4 | 0.957 |
| C8 | C4 | C2 | 4.43E-07 | 9.00E-01 | 7.45E-05 | 9.97E-02 | 1 | C4 | 0.945 |
| C8 | C7 | C2 | 7.78E-01 | 5.09E-02 | 3.73E-09 | 1.71E-01 | 1 | C8 | 0.790 |
| C8 | C7 | C4 | 1.10E-03 | 3.14E-12 | 7.79E-01 | 2.20E-01 | 1 | C4 | 0.813 |
| C8 | C6 | C2 | 1.36E-08 | 1.88E-143 | 2.08E-11 | 1.00E+00 | 1 | C8 | 0.800 |
| C8 | C6 | C4 | 3.15E-22 | 2.19E-149 | 9.65E-01 | 3.53E-02 | 1 | C4 | 0.970 |
| C8 | C6 | C7 | 2.51E-03 | 1.76E-123 | 9.32E-01 | 6.56E-02 | 1 | C7 | 0.933 |
| C8 | C5 | C2 | 4.75E-02 | 1.83E-130 | 1.17E-13 | 9.52E-01 | 1 | C8 | 0.846 |
| C8 | C5 | C4 | 1.99E-08 | 5.78E-123 | 8.73E-01 | 1.27E-01 | 1 | C4 | 0.877 |
| C8 | C5 | C7 | 5.81E-01 | 7.20E-113 | 1.28E-11 | 4.19E-01 | 1 | C8 | 0.795 |
| C8 | C5 | C6 | 1.54E-39 | 9.86E-01 | 2.38E-15 | 1.41E-02 | 1 | C5 | 0.995 |
| C5 | C4 | C2 | 5.18E-137 | 9.82E-01 | 6.09E-07 | 1.85E-02 | 1 | C4 | 0.989 |
| C5 | C7 | C2 | 5.72E-121 | 3.79E-09 | 4.53E-07 | 1.00E+00 | 1 | C2 | 0.766 |
| C5 | C7 | C4 | 1.82E-139 | 1.15E-01 | 1.19E-05 | 8.85E-01 | 1 | C7 | 0.922 |
| C5 | C6 | C2 | 9.46E-01 | 7.30E-08 | 3.64E-08 | 5.38E-02 | 1 | C5 | 0.976 |
| C5 | C6 | C4 | 8.57E-01 | 2.37E-19 | 7.55E-13 | 1.43E-01 | 1 | C5 | 0.891 |
| C5 | C6 | C7 | 9.28E-01 | 9.30E-05 | 4.45E-36 | 7.17E-02 | 1 | C5 | 0.950 |
| C6 | C7 | C4 | 1.40E-140 | 3.62E-01 | 6.10E-01 | 2.81E-02 | 1 | C4 | 0.781 |
| C6 | C7 | C2 | 6.57E-114 | 2.77E-01 | 6.61E-01 | 6.22E-02 | 1 | C2 | 0.851 |
| C6 | C4 | C2 | 5.76E-143 | 9.81E-01 | 1.84E-04 | 1.85E-02 | 1 | C4 | 0.990 |
| C7 | C4 | C2 | 4.02E-01 | 4.25E-01 | 3.90E-05 | 1.73E-01 | 0 | C4 | 0.460 |
