# Supplementary material for: Dynamics of embryonic stem cell differentiation inferred from single-cell transcriptomics show a series of transitions through discrete cell states
Source: eLife. 2017 Mar 15;6:e20487. doi: 10.7554/eLife.20487 (PMC5352225; doi:10.7554/eLife.20487)
Supplement: Figure 4—source data 1. — * The [BMP] and [Aes] modules have the same binary pattern. ** The [FGF] and [WNT] modules have the same binary pattern. DOI: http://dx.doi.org/10.7554/eLife.20487.015 [file elife-20487-fig4-data1.docx]

**Figure 4 – Source Data 1: Gene modules used for modeling the network**

| **[Klf4]** | Ash2l | Esrrb | Eed | Kdm3a | Klf4 | Klf5 | Poldip2 | Tfcp2l1 | Zfp42 |  |
| --- | --- | --- | --- | --- | --- | --- | --- | --- | --- | --- |
|  | Tsc22d1 | Fblim1 | Jarid2 | Atf4 |  |  |  |  |  |  |
| **[Hes6]** | Hes6 | Hmgb2 | Ncl | Zscan10 | Pole | Cbx1 | Prrc2c | Mycn | Rhox5 |  |
| **[Churc1]** | Churc1 | Ttf1 | Cdk9 | Gm13157 | Mtf2 | Pa2g4 | Psmc5 | Ptma | Supt6 | Suz12 |
|  | Tet1 | Klf9 | L3mbtl2 | Cdc5l | Hdac1 |  |  |  |  |  |
| **[Apex1]** | Apex1 | Phc1 | Drg1 | Lin28b | Notch2 | Phb | Plrg1 | Zfp207 | Pcbp1 | Polr2j |
|  | Rpa1 | Baz1b |  |  |  |  |  |  |  |  |
| **[Pax6]** | Pax6 | Fezf2 |  |  |  |  |  |  |  |  |
| **[Atf2]** | Atf2 | Lsm14a | Polr2e | Polr2f | Puf60 | Cbx3 | Mcm3 |  |  |  |
| **[Sox2]** | Chd4 | Dnajc2 | Rbpj | Set | Sox4 | Upf1 | Sox2 |  |  |  |
| **[Baz1a]** | Basp1 | Baz1a | Exoc3 | Fbxo18 | Foxm1 | Med14 | Peg10 | Zfp326 | Tfap2c | Smarcc1 |
|  | Wbp5 | Rbbp7 | Smarcad1 | Zic2 | Trim33 | Psmb6 | Pnn | Rab11a | Zfp91 | Tle3 |
|  | Dnmt1 | Rnf4 | Psip1 | Ing2 |  |  |  |  |  |  |
| **[Msx2]** | Lrrfip1 | Naa15 | Tal2 | Zfp746 | Msx2 | Lbr | Myef2 | Paxbp1 | Tcerg1 | Ep400 |
|  | Zfand6 | Rab18 | Gtf3a | Tipin | Snd1 | Mnat1 | Yy1 | Bptf | Rad50 | Rad51 |
|  | Chd1 | Nono | Hbp1 | Bloc1s1 | Cebpz |  |  |  |  |  |
| **[Snai1]** | Cand2 | Cited1 | Hmga2 | Lef1 | Pdlim4 | Snai1 | Tbx6 | Tead2 | Maged1 | Aebp2 |
|  | Pds5b | Hif1a | Qk |  |  |  |  |  |  |  |
| **[Ciao1]** | Ciao1 | Foxa2 | Keap1 | Msh3 | Tdp2 | Tsg101 | Znrd1 | Polr2c | Pcna | Prkar1a |
|  | Ssbp1 | Phf20 |  |  |  |  |  |  |  |  |
| **[Tead1]** | Tead1 | Lrpprc | Impdh2 | Ezh2 | Snrpb | Sox12 | Gars | Tardbp | Cops5 | Tmpo |
|  | Cdk4 | Edf1 | Ctbp1 | Zyx | Eif3h | Metap2 | Mki67ip |  |  |  |
| **[Hes1]** | Ajuba | Zmat3 | Bbx | Cbx5 | Fam58b | Hes1 | Hmgcs1 | Hmgn5 | Kat7 | Nlrp1a |
|  | Tsn | Rab15 | Rest | Sap18 | Sfmbt2 | Sptbn1 | Sra1 | Taf10 | Tsc22d4 |  |
|  | Wiz | Zfp322a | Zfp593 | Smarca5 | Zfml | Srrt | Erh | Polr2g | Rnf10 | Ctcf |
| **[Oct4]** | Bclaf1 | Utf1 |  |  |  |  |  |  |  |  |
| **[Ets2]** | Polb | Cand1 | Cenpa | Fus | Glyr1 | Gtf2i | Kdm5c | Kif22 | Pbrm1 | Phf5a |
|  | Hmga1 | Preb | Rad51ap1 | Rbms1 | Smarca5 | Top1 | Utp6 | Zmat2 | Zmynd11 | Ets2 |
| **[Hmga1]** | Brd4 | Tial1 | Sall4 | Zfp266 | Sod2 | Son |  |  |  |  |
| **[Sp5]** | Pygo2 | Chtf8 | Dek | Etf1 | Foxo4 | Med10 | Pfdn1 | Pml | Ppp5c | Prkrir |
|  | Sp5 | Qrich1 | Zfp445 | Rrn3 | Sin3b | Ssbp3 | Strn3 | Rab8a | Prrc2a | Pola2 |
|  | Cbfb | Smad4 | Rtf1 | Kdm5b | Rab8a | Lig1 | Yap1 | Topors | Fem1b | Kdm5a |
| **[Otx2]** | Dnmt3b | Trim28 | Crip2 | Aplp2 | Hnrnpu | Fubp1 | Jund | Hmgn1 | Tceal8 | Taf9 |
|  | Hmgb1 | Brcc3 | Zmynd8 | Limd1 | Otx2 |  |  |  |  |  |
| **[T]** | T | Hells | Olig1 |  |  |  |  |  |  |  |
| **[Etv5]** | Etv5 | Gm13154 |  |  |  |  |  |  |  |  |
| **[Smarce1]** | Smarce1 | Strbp |  |  |  |  |  |  |  |  |
| **[Fhl1]** | Fhl1 | Klf6 | Mtpn | Hdgfrp3 | Sox11 | Nr6a1 | Zfp36l1 | Taf13 | Zfand3 | Sox3 |
|  | 0610010K14Rik | Pdlim7 | Hdac2 | Hdac6 | Xbp1 | Ndn | Agfg1 |  |  |  |
| **[Xab2]** | Xab2 | A630089N07Rik | Orc2 | Tceb2 | Fblim1 | Atmin | Cbx7 | Tle4 |  |  |
| **[Gm13051]** | Gm13051 | Taf7 | Gm13251 |  |  |  |  |  |  |  |
| **[Brd7]** | Brd7 | Fah | Khdrbs1 | Zic3 | Litaf |  |  |  |  |  |
| **[Hmgn2]** | Hmgn2 | Ivns1abp | Basp1 | Ctnnb1 | Whsc1 | Top2b | Pdlim5 | Adnp | Btbd1 | Cops2 |
| **[Aes]*** | Dnmt3a | Ctbp2 | Ddx3x | Aes | Foxo1 | Rpa2 | Top2a | Tcea3 | Rab25 | Rad23a |
|  | Tomm6 | Sp1 |  |  |  |  |  |  |  |  |
| **[LIF]** | Cep57 | Lifr | Il6st | Jak2 | Jak3 | Stat1 | Stat3 | Stat5a | Stat5b | Lif |
| **[FGF]**** | Fgf17 | Ctgf | Ctnnb1 | Dstyk | Dusp6 | Fam20c | Fgf1 | Fgf10 | Fgf15 | Fgf16 |
|  | Fgf8 | Fgf18 | Fgf2 | Fgf20 | Fgf21 | Fgf22 | Fgf23 | Fgf3 | Fgf4 | Fgf5 |
|  | Flrt3 | Fgf9 | Fgfbp1 | Fgfbp3 | Fgfr1 | Fgfr2 | Fgfr3 | Fgfr4 | Flrt1 | Flrt2 |
|  | Ndst1 | Frs2 | Frs3 | Grb2 | Hhip | Iqgap1 | Kif16b | Kl | Klb | Lrit3 |
|  | Fgf6 | Nog | Pdgfb | Prkd2 | Rab14 | Runx2 | Setx | Shcbp1 | Sos1 | Trim71 |
|  | Bmp10 | Fgf7 |  |  |  |  |  |  |  |  |
| **[BMP]*** | Bmp8b | Bmp15 | Bmp2 | Bmp2k | Bmp3 | Bmp4 | Bmp5 | Bmp6 | Bmp7 | Bmp8a |
|  | Actr1a | Bmpr1a | Bmpr1b | Bmpr2 | Acvr1 | Acvr1b | Acvr1c | Acvr2a | Acvr2b | Acvrl1 |
|  | Actrt3 | Actr1b | Actr2 | Actr3 | Actr3b | Actr5 | Actr6 | Actr8 | Actrt1 | Actrt2 |
|  | Amer1 | Tgfbr1 | Tgfbr2 | Tgfbr3 |  |  |  |  |  |  |
| **[WNT]**** | Cdh3 | Ankrd10 | Apc | Arntl | Aspm | Bambi | Bcl9 | Bcl9l | Caprin2 | Ccar2 |
|  | Eda | Cdk14 | Cfc1 | Col1a1 | Csnk1d | Csnk1e | Ctdnep1 | Ctnnd2 | Dapk3 | Disc1 |
|  | Fzd9 | Egf | Emd | Folr1 | Fzd10 | Fzd2 | Fzd3 | Fzd4 | Dvl2 | Dvl3 |
|  | Lrrk1 | Gata3 | Gprc5b | Gsk3b | Hoxb9 | Ift20 | Ilk | Ins2 | Kdm6a | Lgr4 |
|  | Otulin | Lrrk2 | Med12 | Mesp1 | Mgat3 | Mitf | Mks1 | Myc | Myh6 | Ndp |
|  | Rspo3 | Plpp3 | Porcn | Prop1 | Psen1 | Pten | Ptk7 | Ptpru | Rab5a | Rnf146 |
|  | Tmem198 | Ryr2 | Sdc1 | Smad3 | Sox7 | Src | Stk11 | Sulf2 | Tbl1x | Tbl1xr1 |
|  | Wnt2b | Tnks | Tnks2 | Trpm4 | Ube2b | Ubr5 | Usp34 | Uty | Vps35 | Wls |
|  | Ccnd1 | Wnt3 | Wnt3a | Wnt7a | Wnt7b | Wnt9a | Wnt9b | Xiap | Zbed3 | Zfp703 |
|  | Dixdc1 | Ccny | Cdc42 | Fzd5 | Fzd7 | Fzd8 | Nfkb1 | Nle1 | Nrarp | Tcf7 |
|  | Tcf7l1 | Dlx5 | Dvl1 | Lgr5 | Lrp5 | Lrp6 | Rnf220 | Rspo1 | Rspo2 | Wnt1 |
|  |  | Tdgf1 | Wnt10b | Wnt2 |  |  |  |  |  |  |

* The [BMP] and [Aes] modules have the same binary pattern.

** The [FGF] and [WNT] modules have the same binary pattern.
