# Supplementary material for: Dynamics of embryonic stem cell differentiation inferred from single-cell transcriptomics show a series of transitions through discrete cell states
Source: eLife. 2017 Mar 15;6:e20487. doi: 10.7554/eLife.20487 (PMC5352225; doi:10.7554/eLife.20487)
Supplement: Figure 4—source data 2. — DOI: http://dx.doi.org/10.7554/eLife.20487.016 [file elife-20487-fig4-data2.docx]

**Figure 4 – Source Data 2:** Binary expression profiles of the gene modules used for modeling the network in the 9 cell clusters

| [Module name] | C_0_ | C_1_ | C_2_ | C_3_ | C_4_ | C_5_ | C_6_ | C_7_ | C_8_ |
| --- | --- | --- | --- | --- | --- | --- | --- | --- | --- |
| [*Klf4*] | 1 | 0 | 0 | 0 | 0 | 0 | 0 | 0 | 0 |
| [*Hes6*] | 0 | 1 | 0 | 0 | 0 | 0 | 0 | 0 | 0 |
| [*Hmga1*] | 0 | 0 | 1 | 0 | 0 | 0 | 0 | 0 | 0 |
| [*Tead1*] | 0 | 0 | 0 | 1 | 0 | 0 | 0 | 0 | 0 |
| [*Sp5*] | 0 | 0 | 0 | 0 | 1 | 0 | 0 | 0 | 0 |
| [*Baz1a*] | 0 | 0 | 0 | 0 | 0 | 1 | 0 | 0 | 0 |
| [*Msx2*] | 0 | 0 | 0 | 0 | 0 | 0 | 1 | 0 | 0 |
| [*Snai1*] | 0 | 0 | 0 | 0 | 0 | 0 | 0 | 1 | 0 |
| [*Ciao1*] | 0 | 0 | 0 | 0 | 0 | 0 | 0 | 0 | 1 |
| [*Churc1*] | 1 | 1 | 0 | 0 | 0 | 0 | 0 | 0 | 0 |
| [*BMP*] | 1 | 1 | 1 | 0 | 1 | 1 | 1 | 1 | 1 |
| [*LIF*] | 1 | 1 | 0 | 0 | 1 | 1 | 1 | 1 | 1 |
| [*FGF*] | 1 | 1 | 1 | 1 | 1 | 1 | 1 | 1 | 1 |
| [*Pou5f1*] | 1 | 1 | 1 | 0 | 1 | 0 | 0 | 0 | 1 |
| [*Sox2*] | 1 | 1 | 0 | 1 | 0 | 1 | 1 | 0 | 0 |
| [*Atf2*] | 1 | 0 | 1 | 1 | 1 | 0 | 1 | 1 | 1 |
| [*Otx2*] | 0 | 1 | 0 | 1 | 0 | 1 | 1 | 0 | 0 |
| [*Smarce1*] | 0 | 0 | 1 | 1 | 1 | 1 | 0 | 1 | 1 |
| [*Ets2*] | 1 | 0 | 1 | 1 | 1 | 1 | 1 | 1 | 0 |
| [*T*] | 0 | 0 | 1 | 0 | 1 | 0 | 0 | 1 | 1 |
| [*Apex1*] | 1 | 0 | 1 | 1 | 1 | 1 | 0 | 1 | 1 |
| [*Hes1*] | 0 | 0 | 1 | 1 | 1 | 1 | 1 | 0 | 1 |
| [*Pax6*] | 0 | 0 | 0 | 1 | 0 | 1 | 0 | 0 | 0 |
| [*Xab2*] | 1 | 1 | 1 | 1 | 0 | 0 | 0 | 0 | 0 |
| [*Gm13051*] | 1 | 1 | 1 | 0 | 0 | 0 | 1 | 0 | 0 |
| [*Brd7*] | 0 | 0 | 1 | 0 | 1 | 0 | 0 | 0 | 1 |
| [*Etv5*] | 1 | 1 | 1 | 0 | 0 | 0 | 0 | 0 | 0 |
| [*Fhl1*] | 0 | 0 | 0 | 1 | 0 | 1 | 1 | 0 | 0 |
| [*Hmgn2*] | 0 | 0 | 1 | 1 | 1 | 1 | 1 | 1 | 1 |
